# The Manipulation of Host Cells by *Salmonella*

**DOI:** 10.3390/ijms27188134

**Published:** 2026-09-12

**Authors:** Micah J. Worley

**Affiliations:** Department of Biology, University of Louisville, Louisville, KY 40292, USA; micah.worley@louisville.edu; Tel.: +1-502-852-0966

**Keywords:** *Salmonella*, gastroenteritis, bacteremia, type III secretion

## Abstract

*Salmonella* remains a serious public health threat. It is estimated to infect over 200 million people per year following inadvertent oral ingestion. It overcomes colonization resistance in part by initially exacerbating host inflammation. *Salmonella* actively invades host cells and extensively manipulates the endocytic pathway to create an intracellular niche conducive to its survival and growth. *Salmonella* counters the inflammation it initially contributes to with the deployment of numerous anti-inflammatory virulence factors to restore host homeostasis, allowing for a longer-term relationship with its host. This review covers the invasion of host cells by *Salmonella* and its intracellular replication and systemic spread strategies. Both the extensive manipulation of intracellular trafficking by some cells as well as escape from the vacuole and growth within epithelial cell cytosol by others are discussed. Also discussed are the typhoid toxin and Vi capsule.

## 1. Introduction

The most studied of the enteric bacterial pathogens is *Salmonella*. This genus is composed of two species: *Salmonella bongori* and *Salmonella enterica*. *S. bongori* is typically a microbe of cold-blooded animals, mainly lizards. *S. enterica* is divided into six subspecies. Many of the nearly 3000 serovars of *S. enterica* subspecies *enterica* are serious pathogens of humans. They cause either gastrointestinal illness, which is usually self-limiting, or a more serious systemic one known as typhoid fever. The typhoidal group is composed of *Salmonella* Typhi, *Salmonella* Paratyphi, and *Salmonella* Sendai, which cause disease only in humans. There are more than 14 million cases of typhoid and paratyphoid fever per year [1]. The common non-typhoidal serovars that infect humans are *S*. Enteritidis, *S*. Typhimurium, and *S*. Dublin. It was estimated by the Centers for Disease Control that in the USA alone, there are 1.3 million infections with these serovars [2]. Of special concern is invasive disease sometimes caused by *S*. Typhimurium and *S.* Enteritidis, which is particularly problematic in sub-Saharan Africa. Bloodstream infections with non-typhoidal *Salmonella* have a fatality rate of 20% following hospitalization [2].

Many of but not all the virulence properties of *Salmonella* are due to its collection of type III effectors. *Salmonella enterica* possesses two independent type III secretion systems, which are encoded by *Salmonella* pathogenicity island-1 and *Salmonella* pathogenicity island-2. In addition to the secretion system itself, each island contains effectors that are injected into host cell cytosol to manipulate the cells in ways that benefit the pathogen. There are also many type III effectors that are injected into host cells by *Salmonella* pathogenicity island-1 or *Salmonella* pathogenicity island-2 and are located outside of the main islands. In total, there are at least 49 type III effectors. Effectors secreted by the type III secretion system encoded within *Salmonella* pathogenicity island-1 primarily affect microbial invasion of host cells and provoke an inflammatory response [3,4,5]. The effectors that are injected by *Salmonella* pathogenicity island-2 promote the intracellular survival and growth of the bacteria by attenuating inflammation and by extensively modifying the *Salmonella*-containing vacuole and enabling systemic dissemination [6,7,8,9,10,11].

### 1.1. Neutralizing Colonization Resistance

Early in infection, *Salmonella* deploys numerous type III effectors that are pro-inflammatory. Typically, the host uses inflammation as a defense mechanism against pathogens. However, *Salmonella* induces and exacerbates inflammation in the intestine as a required component of its virulence [12,13,14]. The induced inflammation diminishes the gut microbiota, which allows *Salmonella* to surmount colonization resistance. The outgrowth of *Salmonella* is promoted by new carbon sources and electron acceptors. The reactive oxygen species generated allow *Salmonella* to grow on ethanolamine by producing tetrathionate, a new respiratory electron acceptor that the microflora cannot use [15,16]. *Salmonella* secretes the *Salmonella* pathogenicity island-1 effector SopE to drive the host to produce nitrate, which is an additional respiratory electron acceptor. Nitrate is a better electron acceptor than tetrathionate and suppresses the expression of genes required for its utilization [17]. S. Typhimurium uses a type VI secretion system to kill commensal bacteria in general and to specifically target *Klebsiella oxytoca*, which is required for *S*. Typhimurium to invade the gastrointestinal epithelium [18].

### 1.2. Invasion of the Gastrointestinal Epithelium

*Salmonella* was one of the first bacterial pathogens observed to invade eukaryotic cells. It resides within host cells in a vacuole termed the *Salmonella*-containing vacuole [19,20,21]. *Salmonella* invades non-phagocytic gastrointestinal epithelial cells by facilitating actin rearrangements and manipulating phosphatidylinositol metabolism in a manner that facilitates membrane ruffling and a macropinocytic event. The small GTPases Rac1, Cdc42, and RhoG are activated by the *Salmonella* pathogenicity island-1 effectors SopE and SopE2 (Table 1) [22,23,24]. SopE2 is found in most serovars of *S. enterica* but is not found in serovar Typhi (Table 2). When GTP-bound, these proteins cause actin to polymerize, which produces ruffles in the membrane and an ensuant macropinocytic event [23,24,25,26]. Activated Rac1 recruits and activates nucleator proteins of the WASP family, including WAVE and N-Wasp, as well as Arp2/3, among others, which results in membrane ruffling and the entry of *Salmonella* [25,26,27]. SopE further activates membrane ruffling through a complex it forms with Rac1 and Caveolin-1 [28]. The *sopE2* gene is present in most strains of *Salmonella*, while SopE is carried by a temperate bacteriophage that is not present in many *Salmonella* isolates [29].

Additionally, SopB, an inositol polyphosphate phosphatase, promotes invasion by facilitating actin cytoskeletal rearrangements [30]. SopB is secreted by the *Salmonella* pathogenicity island-1 type III secretion system. It is widely distributed amongst *Salmonella* serovars. It facilitates *Salmonella* invasion of host cells by altering the host cell membrane’s lipid composition, which is necessary for recruiting host proteins that facilitate entry [31]. The transducing factor SH3-containing GEF is indirectly stimulated by SopB, through its phosphatase activity. It next activates the Rho-family GTPase RhoG, precipitating actin rearrangement [31]. SopB also recruits Rho GTPases, like RhoD, RhoB, RhoH, and RhoJ to the site of invasion with its catalytic activity, activating downstream signaling pathways. Specifically, RhoB and RhoD activation leads to the polymerization of actin which contributes to membrane ruffle formation and the internalization of *Salmonella* by host cells [32]. Also, SopB uses its inositol phosphatase activity to recruit to the plasma membrane SNX18, a SH3-PX-BAR structural domain-sorting nexin protein, altering the phosphoinositide landscape of the membrane. SNX18 recruits Dynamin-2 and N-WASP to the plasma membrane, which is necessary for the invasion of host cells by *Salmonella* and for the formation of *Salmonella*-containing vacuoles [33]. Finally, SopB recruits sorting nexin 9 to the plasma membrane. It plays a role in actin rearrangement and ruffling [34].The *Salmonella* pathogenicity island-1 effector, SipA, is not required for invasion but promotes the process by stabilizing F-actin and decreasing G-actin, which prevents actin filament depolymerization [35,36]. It facilitates *Salmonella* uptake by causing spatial localization of membrane ruffles and outward extension [35,37,38]. SipA is expressed in diverse serovars of *Salmonella*.

### 1.3. Exacerbating Inflammation

By secreting SopB, SopE, and SopE2 into gastrointestinal epithelial cells, *Salmonella* prevents the host from dampening the inflammatory response, which is important for early infection. Alongside Rho-family GTPases, these effectors also activate a non-canonical signaling complex to further exacerbate inflammation [5,23,39]. The *Salmonella* pathogenicity island-1 associated effectors SopA and SopD intensify the inflammatory responses induced by SopE, SopE2, and SopB (Table 3). These two effectors enhance inflammation by targeting innate immune signaling mechanisms to acquire nutrients. Two host E3 ubiquitin ligases, TRIM56 and TRIM65, are targeted by SopA to increase interferon-ß expression though the innate immune receptors MDA5 and RIG-1 [40]. SopD is required for *Salmonella* virulence [41]. It has GTPase-activating protein activity for Rab8, which normally suppresses inflammation [42]. The host-specialized serovars Gallinarum, Pullorum, and Typhi do not express SopA.

SrfA is a novel type III effector, which induces inflammation by promoting NF-κB signaling [43]. It is an ancient protein expressed by most serovars of *Salmonella*. It is secreted into host cells by the *Salmonella* pathogenicity island-1 type III secretion system. It targets the toll-interacting protein, disassociating the IRAK-1-toll interacting protein from IL-1R-associated kinase-1 (IRAK) [44]. The liberated IRAK-1 is phosphorylated and it then activates the NF-κB signaling pathway [44]. This upregulates LPS-induced pro-inflammatory cytokine expression, including IL-8, IL-1β, and TNFα [44]. When *srfA* is deleted, the mutant bacteria are attenuated for virulence, inducing less inflammation, and this results in higher survival rates in infected animals [44,45]. A *srfA* mutant thus has the potential of being a live attenuated vaccine strain [45].

The dominant *sspH2* allele in *S*. Typhimurium is pro-inflammatory [46]. It can be translocated into host cells by the type III secretion system harbored by *Salmonella* pathogenicity island-1 or *Salmonella* pathogenicity island-2. It is primarily secreted by the *Salmonella* pathogenicity island-2 type III secretion system because of its expression pattern [47]. Palmitoylation, a post-translational modification of Cys9, targets SspH2 to the host cell plasma membrane [48,49]. This effector facilitates spatially selective ubiquitination of NOD1 to enhance inflammatory signaling [46]. Interestingly, SspH2 from some strains of *Salmonella* has been reported to be anti-inflammatory. The deletion of *sspH2* from Salmonella *enteritidis* C50336 promotes the expression of IL-1β, IFN-γ, IL-12, and iNOS cytokines [50]. This could be attributable to an allelic difference. In fact, a common allele of *sspH2* in *S*. Dublin has an in-frame 60 bp deletion of one of the leucine-rich repeat domains in the amino-terminus that is important for targeting [51]. This suggests it may act on different proteins than the dominant allele from *S.* Typhimurium. This is interesting, considering that *S.* Dublin is primarily a systemic pathogen that does not induce significant inflammation. SspH2 is not present in serovars Choleraesuis, Gallinarum, and Pullorum but is found in serovar Typhi. More work is required to understand the effects of the different alleles of *sspH2* on *Salmonella* virulence.

### 1.4. Escape from the Salmonella-Containing Vacuole

*Salmonella* sometimes escapes the vacuole that contains it and proliferates extensively within the cytosol of epithelial cells [52,53,54]. In fact, as early as 15 min post-invasion, some of the internalized bacteria can be detected in epithelial cell cytosol [55]. By one hour post-infection, up to 30% of internalized bacteria are free within the cytosol [55,56,57]. Ultimately, *Salmonella* hyper-replicates in the epithelial cell cytosol but not within the macrophage cytosol [53,54,58]. Interestingly, the amount of bacterial replication within epithelial cell cytosol far exceeds that observed within the *Salmonella*-containing vacuoles [53,54]. Cytosolic and vacuolar *Salmonella* have unique gene expression programs that are induced by their intracellular environment. Genes that are usually associated with bacteria outside of host cells, such as *Salmonella* pathogenicity island-1 and *Salmonella* pathogenicity island-4 are preferentially expressed in the cytosol versus the vacuole [59]. Two *Salmonella* pathogenicity island-1 effectors, SopB and SipA, are necessary for efficient colonization of epithelial cell cytosol, firmly implicating this pathogenicity island in a post-invasion role [60]. When a multigene deletion library was screened in epithelial cells, three genes, *ydgT, recA*, and *corA* were found to normally promote cytoplasmic *Salmonella* proliferation, while one gene, *asmA*, was implicated in reducing replication within the cytosol (Table 4) [61]. None of these genes were required for vacuolar replication or lysis of the vacuole, indicating that they specifically regulate microbial proliferation within the epithelial cell cytosol [61].

CorA is the primary Mg2^+^ influx channel in *S*. Typhimurium indicating a requirement for Mg2^+^ uptake for bacterial growth within the cytosol. Interestingly, CorA was implicated as a putative determinant for intestinal colonization in chickens, cows, and pigs [62]. CorA is required for systemic infection of BALB/c mice following either oral or intraperitoneal infection [63]. RecA is necessary for recombinatorial DNA repair [64]. The host cell cytosol may be a general site of bacterial DNA damage, as *Fransicella tularens* is mutants that are defective in DNA repair have replication defects within the cytosol of U937 human macrophages [65]. The third gene shown to be needed for cytosolic replication of *S*. Typhimurium is *ydgT*. A *ydgT* mutant replicates within the *Salmonella*-containing vacuole at wild-type level but grows very poorly in epithelial cell cytosol. YdgT and its paralogue, Hha, act as negative regulators of horizontally acquired genes such as *Salmonella* pathogenicity island-1, *Salmonella* pathogenicity island-2, and *Salmonella* pathogenicity island-5, through a poorly characterized mechanism [66,67,68]. In a genetic screening, Δ*asmA* was the only mutant identified that proliferated more in epithelial cell cytosol than wild-type bacteria [61]. Deleting *asmA* enhanced IL-18 release from epithelial cells, presumably due to enhanced inflammasome activation [61]. The increased number of cytosolic bacteria may be attributable to a reduced ability to induce pyroptotic cell death and exit epithelial cells. YdgT, RecA, CorA, and AsmA are expressed by diverse serovars of *Salmonella*.

### 1.5. The Salmonella-Containing Vacuole

The *Salmonella* that remains within the vacuoles creates a unique compartment within host cells termed the *Salmonella*-containing vacuole [69,70,71,72]. *Salmonella*-containing vacuole biogenesis is accomplished with *Salmonella* pathogenicity island-1 and *Salmonella* pathogenicity island-2 effectors and the recruitment of multiple small Rab GTPases [73,74,75]. The biogenesis of the *Salmonella*-containing vacuole occurs in three temporal phases: early biogenesis, 10 min to 1 h post-infection, intermediate, 1–4 h, and late, over 4 h. Within minutes of its biogenesis, early endosomes interact with the *Salmonella*-containing vacuole. The type III effectors SopE and SopB recruit the small GTPase Rab5 to the *Salmonella* containing-vacuole. This promotes the fusion of early endosomes with the *Salmonella*-containing vacuole. An effector downstream of Rab5, VPS34/p150, which is a phosphatidylinositol 3-kinase, is recruited and generates PtdINs(3)P [76]. Importantly, the SopB phosphatase causes PI3P to accumulate on early *Salmonella*-containing vacuoles, causing Rabs and early endosome antigen 1 to bind to them. This prevents *Salmonella*-containing vacuoles from fusing with lysosomes. It also results in the recruitment of SNX1 and SNX3. These sorting nexins are important for dynamic tubule formation, referred to as spacious vacuole-associated tubules [77,78,79,80]. Over the next hour, the *Salmonella*-containing vacuole develops into a late endosome. Rab5 is lost and Rab7, lysosomal associated membrane protein 1 and 2, and v-ATPase are recruited, which requires SNX1 and SNX3 [78,81,82,83]. The *Salmonella* pathogenicity island-1 secreted type III effector SopF binds to phosphoinositide to promote the stability of the *Salmonella*-containing vacuole [84]. SipA prevents the maturation of *Salmonella*-containing vacuoles into lysosomes by mimicking an R-SNARE and recruiting Syntaxin8, thereby promoting the fusion of early endosomes with the *Salmonella*-containing vacuole [85].

Cytoplasmic dynein and Rab7 facilitate the *Salmonella*-containing vacuole transiting from the invasion site to the perinuclear microtubule organizing center in the intermediate stage of development. GTP-bound Rab7 recruits the effector RILP, which brings the *Salmonella*-containing vacuole close to the microtubule organizing center and the Golgi apparatus [86]. Most *Salmonella*-containing vacuoles are clustered in this region during the initial rounds of bacterial replication. The recruited v-ATPase acidifies the *Salmonella-*containing vacuole, which allows the *Salmonella* pathogenicity island-2 encoded type III secretion system to bind to the membrane and subsequently secrete effectors into the cytoplasm [87]. At a later stage of infection, highly dynamic tubules know as *Salmonella*-induced filaments emerge from the *Salmonella-*containing vacuole. The *Salmonella* pathogenicity island-2-secreted effectors SifA, SseK3, SteA, SseG, SseF, SseJ, PipB2, SopD2 are involved in the formation of *Salmonella*-induced filaments, while SpvB has a negative effect. Two *Salmonella* pathogenicity island-2 effectors, SseF and SseG, bind the Golgi resident protein ACBD3, which anchors the *Salmonella*-containing vacuole at the Golgi apparatus in infected epithelial cells, which enables bacterial replicaton [88]. They counter the effectors SteA and PipB2, causing microtubule motors to drive the vacuole away from the Golgi [88,89,90]. Two effectors, PipB and PipB2, localize to lipid rafts in the membranes of *Salmonella*-induced filaments and *Salmonella*-containing vacuoles [91]. The conservation of these two effectors amongst the various serovars of *Salmonella* suggests that they contribute to virulence in different hosts. PipB2 contributes to the biogenesis of tubules that emanate from the *Salmonella*-containing vacuole [92]. The autoinhibition of the kinesin-1 molecular motor is relieved by PipB2 [92]. SopD2 interferes with the ability of Rab7 to bind the effectors FYCO1 and RILP, which prevents endosomes from delivering cargo to lysosomes. SifA, SteA, SseG, SseF, and PipB2 are present in diverse serovars of *S*. *enterica* while SseK3 is absent from Gallinarum, Pullorum, and Typhi and SseJ and SopD2 are not expressed by Typhi.

SifA stabilizes the *Salmonella*-containing vacuole and is necessary for *Salmonella*-induced filament formation in infected epithelial cells [93,94]. Within the *Salmonella*-containing vacuole or SIF continuum, *Salmonella* has increased metabolic activity compared to intra-vacuolar *Salmonella* that is not associated with SIFs [95]. SifA thus helps *Salmonella* overcome nutritional restriction by facilitating the acquisition of nutrients from the host-cell endosomal system [95]. Additionally, SifA reduces lysosomal antibacterial activity by subverting Rab9-dependent retrograde trafficking of the mannose-6-phosphate receptor by binding SKIP/PLEKHM2, which reduces lysosomal antibacterial activity [96]. SifA in complex with SKIP interacts with HOPS (homotypic fusion and protein sorting) complex subunit Vps39 to facilitate recruitment of this tethering factor to the *Salmonella*-containing vacuole [97]. *Salmonella* thus gains access to host membrane and nutrients by recruiting endosomal and lysosomal membrane fusion machinery to the vacuole it resides within, thereby ensuring intracellular survival and replication [97].

SseJ is an effector that is secreted by *Salmonella* pathogenicity island-2. It is usually not present in strains of *Salmonella* that cause extraintestinal disease [98]. It regulates membrane dynamics of the *Salmonella*-containing vacuoles. It participates in the biogenesis of SIFs [99,100]. It localizes to the cytoplasmic side of the *Salmonella*-containing vacuole where, along with SseL, it promotes vacuolar integrity by recruiting the eukaryotic lipid transporter oxysterol-binding protein 1 [101]. It stabilizes the vacuole by altering its lipid and protein content. SseJ is activated through its interaction with GTP-RhoA to have glycerophospholipid–cholesterol acyltransferase activity [99,102,103]. SseJ suppresses the expression of the host cholesterol transporter ABCA1 by activating a signaling cascade that involves the host kinases FAK and Akt [104]. Inhibiting Abca1 to facilitate the accumulation of cholesterol during infection is important for intracellular survival [104]. SseL is found in all serovars of *S*. enterica whereas SseH is absent from serovar Typhi.

The *Salmonella* pathogenicity island-2 effector SseF is required for proper positioning of the *Salmonella*-containing vacuole [105]. SseF is necessary for the recruitment to the *Salmonella*-containing vacuole of dynein and the formation of clusters of replicating bacteria [105]. In the C-terminal half of SseF are three transmembrane helices that are required for the intracellular positioning of the *Salmonella*-containing vacuole to a juxtanuclear, Golgi-associated localization [105]. Thus, SseF, SseG, and SifA enable precise positioning of the *Salmonella*-containing vacuole by affecting the recruitment of microtubule motor proteins [105]. *Salmonella* also injects SseF into host cells to detoxify the *Salmonella*-containing vacuole. SseF promotes intra-vacuolar proliferation by interacting with RAB7 and the mitochondrial citrate carrier [106]. The mitochondrial citrate carrier regulates local citrate levels and reduces oxidative stress by attenuating the production of reactive oxygen species [106]. *Salmonella* thereby evades host antimicrobial defenses by hijacking a mitochondrial metabolite transporter to influence the vacuolar environment and evade antimicrobial defenses [106]. Interestingly, pharmacological inhibition of the mitochondrial citrate carrier sensitizes *Salmonella* to anti-microbial defenses [106]. Thus, the mitochondrial citrate carrier may potentially serve as a host target for antimicrobial therapy. SseF is conserved amongst serovars of *S. enterica*.

*Salmonella* exploits the host glucose transporter Glut1 to the pathogen’s advantage. First, *S*. Typhimurium activates the MAPK signaling cascade with *Salmonella*-pathogenicity island-1 to upregulate Glut1 to enhance host uptake of glucose [107]. Secondly, the bacteria recruit Glut1 to the vacuolar membrane, thereby creating a glucose-import conduit that facilitate bacterial acquisition of host glucose from the cytosol [107]. Finally, in a newly discovered regulatory mechanism that potentiates Glut1 transport, the bacteria cause K29-linked ubiquitination on the membrane of the *Salmonella*-containing vacuole [107]. It is interesting to consider that *Salmonella* pathogenicity island-1 effectors initiate early MAPK activation, which stimulates Glut1 expression to support the initial survival of *Salmonella* [107,108]. However, prolonged MAPK activation might lead to host cell death or excessive inflammation. *Salmonella* counters the early MAPK activation with the *Salmonella* pathogenicity island-2 effector SpvC, which is a phosphothreonine lyase that irreversibly inactivates MAPKs [109,110]. This attenuation suppresses pro-inflammatory and pro-apoptotic gene expression, creating a permissive niche for bacterial replication and also allows for extraintestinal dissemination [11,111,112,113].

S. Typhi is exquisitely host-adapted. It can only infect humans while the closely related S. Typhimurium has a broad host range. S. Typhimurium promotes its proliferation within mouse macrophages with SopD2 and GtgE, which *S*. Typhi lacks. Deleting either gene attenuates *S*. Typhimurium virulence in mice [114,115]. Both effectors target Rab32 to prevent the delivery to the *Salmonella*-containing vacuole of anti-microbial factors. Interestingly, when *S*. Typhi is engineered to express either of these two effectors, which it normally lacks, Rab32 recruitment is interdicted and the bacteria can grow in murine as well as human macrophages [115]. Thus, the strict host specificity of *S*. Typhi is determined by the lack of a single effector.

The *Salmonella* pathogenicity island-2 effector SrfH was reported to affect infected host cell adhesion and motility [9,10,116,117,118]. Interestingly, different alleles of *srfH* have different effects on the adhesive and migratory properties of cells [10]. A separate function of SrfH is to acquire host cholesterol for the *Salmonella*-containing vacuole. It facilitates peroxisome-lysosome interaction and mediates the transfer of lysosomal cholesterol to *Salmonella*-containing vacuoles, using peroxisomes as a bridge [119]. SrfH has a variant of the PTS1 peroxisome-targeting sequence, GKM [119]. It can localize to the peroxisomes where it activates ARF-1, which leads to the recruitment of phosphatidylinosiotol-5-phosphate-4 kinase, which generates phosphatidylinosiotol-4-5-biphosphate on peroxisomes [119]. This enhances peroxisome/lysosome interaction and allows for the transfer of lysosomal cholesterol to *Salmonella*-containing vacuoles. This cholesterol facilitates bacterial growth by enhancing *Salmonella*-induced filament integrity as well as the stability of *Salmonella*-containing vacuoles [119].

### 1.6. Limiting Inflammation

It is easy to overlook that host–pathogen interactions have been shaped by long-standing associations that do not maximize pathogen replication at all costs but rather seek to maintain infection while preserving host homeostasis. *Salmonella* in fact has numerous mechanisms not just to induce inflammation but also to attenuate it. Restoring host homeostasis allows *Salmonella* to establish a longer-term association with the host than it otherwise would. Inflammation is also dampened by some strains to facilitate systemic spread after overcoming colonization resistance [11]. If fact, deleting some virulence factors results in less intestinal disease but facilitates bacterial spread to systemic sites.

*Salmonella* deploys numerous type III effectors with anti-inflammatory properties. SopE, in addition to its role in activating Rac1 to promote actin polymerization, can cause depolymerization of actin through PLCϒ and villin as part of this effector’s dynamic remodeling of the cytoskeleton [120]. Excessive actin polymerization can further be prevented by SopA, which inhibits Cdc42 activation (Table 5) [121]. SopA thus results in a self-limiting invasion process to avoid excessive cellular damage and to prevent over-stimulation of an immune response.

SteE/SarA/GogC is secreted by both *Salmonella* pathogenicity island-1 and *Salmonella* pathogenicity island-2. This effector essentially reprograms innate immunity by triggering the generation of an anti-inflammatory response [122,123]. It converts the mammalian serine/threonine kinase GSK3 into a tyrosine kinase to direct macrophage polarization. GSK3 phosphorylates this effector, which then reprograms it to phosphorylate a tyrosine residue on signal transducer and activator of transcription 3 (STAT3) [122,123]. The substrate and amino acid specificity of GSK3 are thus reprogrammed by SteE. Activated STAT3 and GSK3 allow SteE to upregulate the M2 macrophage marker interlukin-4Rα [122,123]. SteE thus induces an anti-inflammatory environment by reprogramming innate immunity [122,123]. SteE is not found in serovars Enteritidis or Typhi.

GogB is a type III effector harbored by the Gifsy-1 temperate bacteriophage in *S*. Typhimurium that can be secreted by *Salmonella* pathogenicity island-1 or *Salmonella* pathogenicity island-2 [124]. It can also be found in some strains of serovar Enteritidis and in serovar Dublin. It acts as an anti-inflammatory agent to limit host tissue damage. It accomplishes this by interfering with the NF-κB signaling pathway [125]. It targets the host SCF E3 type ubiquitin ligase by interacting with Skp1 and the human F-box only 22 protein [125]. It downregulates inflammation-enhanced colonization by inhibiting IκB degradation and decreasing NF-κB activation [125]. GogB is thus an anti-inflammatory effector that attenuates inflammation-enhanced colonization to limit tissue damage during infection.

AvrA is also involved in attenuating the host’s inflammatory response. After being secreted by the type III secretion system encoded by *Salmonella* pathogenicity island-1 or *Salmonella* pathogenicity island-2, AvrA diminishes intestinal inflammation and systemic cytokine responses by inhibiting the JNK and NF-κB pathways [126,127]. It is not found in serovars Choleraesuis or Typhi. AvrA prevents the degradation of IκBα by deubiquinating it, which shields it from degradation, which inhibits NF-κB activation [126,127]. It diminishes inflammation of the intestine and the apoptosis of epithelial cells and systemic cytokine responses [126,127]. It diminishes the proapoptotic innate responses to *Salmonella* in the mouse intestinal mucosa [127]. By diminishing immune responses, it helps *Salmonella* establish an intracellular niche. The activity of AvrA is consistent with *Salmonella* disease in mammalian hosts, where the bacteria elicit temporally regulated inflammation but do not destroy epithelial cells.

*Salmonella* induces intestinal inflammation by potently activating the NF-κB signaling pathway, which is required for acquiring nutrients in the gut [5,15,25,108,128]. However, all *Salmonella* isolates encode at least one of the zinc metalloproteases GogA, PipA, or GtgA, which counter inflammation. GogA and GtgA can be secreted by the type III secretion system harbored by either *Salmonella* pathogenicity island-1 or *Salmonella* pathogenicity island-2, while PipA is only injected into host cells by the *Salmonella* pathogenicity island-1 secretion system. The conservation of these effectors reveals the importance of reigning in the inflammation that *Salmonella* initially provokes. Notably, when a strain was engineered to lack these three effectors, virulence and lethality were heightened [129]. This is likely attributable to an increase in pro-inflammatory cytokines in the gut. These three effectors cleave the DNA binding loop of the NF-κB transcription factors p65 and RelB [129]. These effectors are thus proteases that cleave central components of the NF-κB signaling cascade to diminish virulence to preserve the host [129].

SseK1, SseK2, and SseK3 form a family of effectors that are secreted by the type III secretion system encoded by *Salmonella* pathogenicity island-2 that manipulate NF-κB signaling. They inhibit TNFα-stimulated NF-κB signaling in a manner that requires N-acetylglucosamine transfer to arginine residues [130,131]. This requires a DXD motif in SseK1 and SseK3 [130]. During infections of macrophages, SseK proteins prevent TNFα-induced cell death [130]. SseK1 and SseK3 have different substrate specificities [130]. SseK1 results in the GlcNAcylation of FADD and TRADD, which are death domain-containing proteins [130]. SseK3 results in weak GlcNAcylation of TRADD but not FADD [130]. Unidentified substrates likely account for the additive phenotype of *Salmonella* that lacks both SseK1 and SseK3 [130]. SseK1 is not present in Gallinarum or Typhi, while SseK2 is only present in Typhimurium. SseK3 is present in some strains of Enteritidis and Typhimurium as well as Dublin and Choleraesuis but not in Gallinarum, Pullorum, or Typhi.

SteA is secreted by the type III secretion system encoded by *Salmonella* pathogenicity island-1 or *Salmonella* pathogenicity island-2. It localizes to the plasma membrane of infected cells by binding phosphatidylinositol 4-phosphate [132]. SteA suppresses the degradation of the inhibitor of NF-κB, IκB [133]. This effector thus suppresses proinflammatory responses of the host [133]. Mice infected with *steA* mutant bacteria die sooner than those infected with wild-type *Salmonella*. This suggests that SteA dampens the inflammatory response. Cells infected with *steA* mutant bacteria have fewer SIFs than those infected with wild-type bacteria. It may affect microtubule motors on *Salmonella*-containing vacuoles [134]. The *Salmonella*-containing vacuoles are clustered when infected with a *steA* mutant and contain more bacteria than when infected with wild-type bacteria [134]. It is functionally linked to SseF and SseG [134]. SteA is broadly distributed amongst *S*. *enterica* serovars.

SspH1 is a type III effector located outside of *Salmonella* pathogenicity island-1 and *Salmonella* pathogenicity island-2 [48]. It is a leucine-rich repeat protein with novel E3 ligase activity, which is only found in serovar Typhimurium. The amino terminal gives it specificity towards ubiquitination targets, while the carboxyl terminal region of the protein has E3 ligase activity [135]. SspH1 can be secreted by the type III secretion system encoded by *Salmonella* pathogenicity island-1 or *Salmonella* pathogenicity island-2 but is primarily injected by *Salmonella* pathogenicity island-2 because of its expression pattern. SspH1 does not share sequence similarity or have similar structure to eukaryotic E3s. SspH1 is an anti-inflammatory protein that acts in the nucleus [136,137]. It targets PKN1, which is a host serine-threonine protein kinase, to prevent NF-κB-dependent gene expression [136,137].

The *Salmonella* plasmid virulence (*spv*) operon is important for human pathogenesis. The *spv* operon is expressed by diverse non-typhoidal serovars of *Salmonella* but is not contained by serovar Typhi. SpvB, SpvC, and SpvD are primarily, although not exclusively, secreted into host cell cytosol by the *Salmonella* pathogenicity island-2 type III secretion system. SpvB is an ADP-ribosylating toxin, which is essential for *Salmonella* virulence in mice [138]. Beyond destabilizing the cytoskeleton, SpvB is also associated with the enhanced survival and intracellular replication of *Salmonella*. It inhibits NF-κB activity by downregulating IKKβ by targeting KEAP1 to delay the host immune response thereby facilitating intracellular bacterial survival and proliferation [139]. It also interferes with intracellular iron homeostasis via regulation of the transcription factor NRF2 [140]. SpvB modulates the hepcidin-ferroportin axis, which regulates systemic iron metabolism. SpvB increases hepatic hepcidin synthesis, which reduces the cellular iron exporter ferroportin, which counters the limitation of iron availability [141]. The amino terminus of SpvB interacts with clathrin and AP-1, one of its adaptors to subvert host membrane trafficking [142]. It inhibits host endocytosis and protein secretion. AP-1-transported substrates are believed to play a role in restricting pathogen proliferation [142].

SpvC is a phosphothreonine lyase that dampens inflammation by irreversibly dephosphorylating Erk1/2, p38, and JNK. This attenuates the production of pro-inflammatory cytokines, such as TNFα and IL-8, helping to restore the infected host cell to homeostasis [109,110,143,144]. SpvC inhibits pyroptosis as well as autophagy [113]. It suppresses NLRP3 as well as NLRC4 [113]. A *spvC* mutant displays no defect versus wild-type bacteria for growth within macrophages but is incapable of reaching the bloodstream [11,145]. Thus, SpvC seems to dampen the inflammatory response of infected cells enabling systemic dissemination of phagocytes harboring *Salmonella* [11,116]. Strains of *S*. Typhimurium lacking either SpvC or AvrA cause more severe intestinal inflammation than wild type bacteria in mouse models. Thus, both effectors help the bacteria evade immune detection [112,126].

SpvD plays a role in virulence during systemic infection of mice [146,147]. SpvD interferes with the nuclear transport machinery of host cells to manipulate its immune response. SpvD manipulates the exportin Xpo2, which is involved in nuclear-cytoplasmic recycling of importins [147]. This interaction attenuates the recycling of importin-α from the nucleus, which leads to a defect in p65 nuclear translocation, which prevents the activation of NF-κB regulated promoters [147]. Although *spvD* is conserved amongst various serovars of *S*. enterica, a single nucleotide polymorphism separates the major allele found in serovar Typhimurium from the major allele in serovar Enteritidis [148]. Interestingly, this polymorphism changes the hydrolytic activity of SpvD and the amount of inhibition of the NF-κB pathway and the degree of bacterial virulence in mice [148].

Following cellular ruffling through which *Salmonella* enters host cells, the cells rapidly recover. This is due to temporal regulation of *Salmonella* virulence effector function by proteasome-dependent protein degradation [149]. The bacterial encoded GEF SopE and the GAP SptP are required for host cell invasion by *Salmonella*. Both effectors are delivered to host cell cytosol by the type III secretion system encoded by *Salmonella* pathogenicity island-1. Both virulence factors are secreted into host cells in similar amounts early in infection; however, the proteasome rapidly degrades SopE, which stimulates ruffling. SptP on the other hand, has much slower degradation kinetics [149]. SptP acts as a GTPase-activating protein for Rac and Cdc42, which shuts the proteins off after they were initially activated by SopB and SopE/SopE2 [150]. Because SptP has a longer half-life than SopE/SopE2, the ruffling is transient in nature and this allows the host cell to return to homeostasis [149]. SptP is conserved amongst *Salmonella* serovars.

SopB and SopD are both effectors that possess opposed functions. SopD, which is required for virulence, is a *Salmonella* pathogenicity island-1 effector that can stimulate both pro- and anti-inflammatory signaling by targeting an anti-inflammatory pathway orchestrated by the Rab8 GTPase [151,152,153]. SopD shows GTPase activating protein activity towards Rab8 and thus stimulates inflammation by inhibiting this GTPase [42]. SopD also stimulates an anti-inflammatory signaling cascade by displacing Rab8 from its cognate guanosine dissociation inhibitor [42]. Thus, remarkably, this bacterial effector protein has evolved to exert both agonistic and antagonistic activities towards the same host target to either enhance or suppress the inflammatory response to *Salmonella*. In addition to activating Cdc42 and stimulating downstream pro-inflammatory signaling, SopB has anti-inflammatory effects with its phosphatidylinositol phosphatase activity. SopB activates Akt through a signaling cascade the involves PI3k, PDK1, and mTORC2. This leads to the phosphorylation of Yes-associated protein, which inhibits the NLRC4 inflammasome in B cells. This results in a host cell environment that promotes bacterial survival [154].

### 1.7. Virulence Factors That Promote Salmonella Spread Within a Host

*Salmonella* infections are generally mild provided they are contained with the gastro intestinal tract. Such infections rarely require hospitalization. However, when bloodstream infections occur, they are frequently fatal. Thus, the transition from the gut to the blood is an area in need of additional study considering the potential for thwarting more serious infections. A major, mostly overlooked role for *Salmonella*-pathogenicity island-2 effectors may be the exploitation of the adhesive and migratory properties of infected phagocytes. Mutations of the *Salmonella* pathogenicity island-2 type III secretion system show modest defects in intracellular growth in cell culture models of infection, while they are completely avirulent in animals [6,7,8,155,156]. In fact, not a single *Salmonella* pathogenicity island-2 type III secretion system structural mutant reached the bloodstreams of animals at any oral inoculum tested [156,157,158]. This suggests that a major role of *Salmonella* pathogenicity island-2 may be to promote intra host dissemination. In fact, at least four *Salmonella* pathogenicity island-2 type III effectors, and one secreted by *Salmonella* pathogenicity island-1, are involved in the transition of *Salmonella* from gastrointestinal to systemic disease.

*Salmonella* gains access to the lamina propria by injecting the *Salmonella* pathogenicity island-1 effector SopF into gastrointestinal epithelial cells, which triggers necroptosis [159]. SopF promotes the systemic spread of *Salmonella* by manipulating PANoptosis [159]. Intestinal epithelial cell panoptosis (pyroptosis, apoptosis and necroptosis) is a pivotal host defense for compartmentalizing foodborne pathogens. Pyroptosis and apoptosis result in the expulsion of infected epithelial cells, which protects the host from systemic dissemination of bacteria [159,160,161]. SopF downregulates caspace-8, which inhibits pyroptosis and apoptosis while promoting necroptosis [159]. SopF-triggered necroptosis allows the infection to proceed to the lamina propria and ultimately, the bloodstream and deeper tissue [159]. SopF is conserved amongst diverse *Salmonella* serovars.

The type III effector SrfH has many different naturally occurring alleles, at least two of which affect the dissemination of *Salmonella*-infected macrophages within its host. Intriguingly, these two alleles have opposite effects [9,10,116,162]. SrfH Asp103 is the dominant allele in non-typhoidal *Salmonella*. Cys178 His216 and Asp231 are three catalytic resides in the C-terminus of SrfH that deamidate the heterotrimeric G protein Gαi2 [117,118]. This prevents infected macrophages that send processes through the gastrointestinal epithelium to engage in antigen sampling by disassociating from the epithelium and then reverse transmigrating through the blood vascular endothelium [10]. SrfH Asp103 also reduces the migration of *Salmonella*-infected phagocytes to the regional lymph nodes [163]. Of those that make it to systemic organs in mice, this allele discourages macrophages from following T-cell chemoattractive gradients [117]. Interestingly, this dominant allele usually prevents strains from causing systemic infections in humans [51].

Of further interest, the minor *srfH* allele SrfH Gly103 accelerates the deadhesion/productive migration of infected macrophages through an interaction with the host protein TRIP6 [9,10]. SrfH Asp103 is pseudogenized in S. Typhimurium ST313 in Sub-Saharan Africa but is expressed in ST313 present in South America and the UK. This is interesting as there are only three genes that have been pseudogenized in ST313 lineage 2, responsible for epidemic *S*. Typhimurium bloodstream infections in Sub-Saharan Africa, versus ST313 circulating in the UK [164]. ST313 in the UK is exclusively associated with gastrointestinal infections. Which allele, if any, of *srfH* a strain carries may be key to whether an infection is strictly gastrointestinal or alternatively disseminates to the blood.

The major *srfH* allele found in *S*. Dublin, which causes invasive disease, substitutes a threonine residue for a proline at residue 213 [51]. This is intriguing as this single-nucleotide polymorphism changes a residue that is three positions away from of the conserved residues in the catalytic triad in the C-terminus. Perhaps this *srfH* allele does not confine infections to the gastrointestinal tract. SrfH is absent from nearly all strains of *S*. Typhi [51]. *S.* Typhi may spread systemically through different pathways than non-typhoidal *Salmonella* or alternatively may possess a functional analog of SrfH that promotes systemic spread.

Many members of non-typhoidal *Salmonella* carry the virulence plasmid, which contains the *Salmonella* virulence plasmid (*spv*) operon [165]. Strains isolated from the blood of patients with non-typhoidal *Salmonella* infections almost always express the *spv* operon. SpvC does not promote the survival or proliferation of *Salmonella* in macrophages but *spvC* mutants are incapable of colonizing the bloodstream [109,145]. SpvC dephosphorylates the host proteins p38, JNK, and Erk1/2, thereby dampening the inflammatory response. This reduces the inhibition of cellular migration normally mediated by the cytokine macrophage migration inhibitory factor (MIF). A reduction in MIF may allow infected macrophages to traverse the blood vascular endothelium.

Different alleles of *spvD* have different effects on the systemic spread of *Salmonella* [147,148]. SpvD is highly conserved amongst non-typhoidal *Salmonella*. However, near the catalytic triad, residue 161 of the dominant allele in serovar Typhimurium is an arginine whereas the dominant allele in serovar Enteritidis has a glycine in this position [148]. SpvD Gly161 more strongly suppresses NF-κB immune responses than SpvD Arg161, which results in greater systemic dissemination in orally infected mice [147,148]. One study reported that Δ*spvBCD* does not affect the intracellular survival of *S*. Typhimurium but that it is unable to enter the blood of mice at any time point following oral infection [11,109,145]. All the numerous effectors with anti-inflammatory properties need to be tested for the ability to accelerate the systemic spread of infection.

SteC is the only *Salmonella* effector that is a kinase [166,167]. *Salmonella* secretes it into infected macrophage cytosol where it phosphorylates Ser19 and Thr20 of the myosin light chain protein My112a [168]. SteC can use all four of the NTPs as phosphate donors, which allows it to outcompete host kinases, which use ATP [168]. The modification of My112a precipitates actin rearrangement, which results in macrophage migration and reverse transmigration through the blood vascular endothelium [168]. SrfH and the *spv* operon are typically not found in typhoidal *Salmonella*. However, SteC is present in typhoidal as well as non-typhoidal *Salmonella* serovars.

### 1.8. The Typhoid Toxin and Vi Capsule

S. Typhi causes typhoid fever, an often-fatal systemic disease that has plagued humans throughout our history. It remains a major public health threat, causing disease in 10.9 million people annually, killing around 200,000 per year [1,169,170,171,172,173]. Bacterial genotoxins damage host DNA and thus activate DNA damage response pathways. The typhoid toxin, which is primarily found in *S.* Typhi and *S*. Paratyphi A, is a pyramid composed of five PltB molecules at the base and one molecule of PltA and CdtB at the top. The A_2_B_5_ exotoxin is produced by *S*. Typhi within the *Salmonella*-containing vacuole and is secreted into the lumen of the vacuole by a phage-derived pathway classified as a type 10 secretion system [174]. The host’s CI-M6PR/COPII trafficking machinery sorts the toxin for export [174]. It enters target cells through retrograde transport to the ER [174]. Typhoid toxin brings together an ADP ribosyltransferase (PltA) with a DNase I-like nuclease (CdtB) on a pentameric delivery ring of PltB. In a *S.* Typhi human challenge model, when volunteers were infected with either wild-type *S*. typhi or a derivative in which the typhoid toxin was deleted, no difference in typhoid infection was observed [175]. This indicates that typhoid toxin is not necessary for infection and early typhoid fever. The experiment did not look at the role of the typhoid toxin in severe disease or long-term bacterial carriage for safety considerations. However, intriguingly, when *S*. Typhimurium was engineered to express functional typhoid toxin or an inactive version and mice were infected with the strains, typhoid toxin promoted host survival [176]. Inclusion of an active version of the toxin intriguingly increased the frequency of asymptomatic carriers [176]. This suggests that typhoid toxin may lead to bacterial persistence by interfering with the ability of immune cells to clear the infection.

S. Typhi has 601 genes on 82 genetic islands that are absent from *S.* Typhimurium. *Salmonella* pathogenicity island-7 is the largest of these islands. It carries nine genes (*tviB-*E and *vexA-E*) that encode the Vi capsular polysaccharides (CPSs)’ biosynthesis apparatus. The biosynthesis system is a critical virulence factor of numerous bacterial pathogens and is used in injectable typhoid vaccines [177,178,179,180,181,182,183,184,185,186]. The Vi capsule of *S*. Typhi provides it with protection from the innate immune response by deterring neutrophil phagocytosis and interfering with the deposition of complement [187,188,189]. Interestingly, single missense mutations in Vi capsule synthesis genes confer hypervirulence to *S*. Typhi. Hypo Vi capsule variants are distributed world-wide, while hyper Vi variants are most prevalent in African countries [190].

## 2. Discussion

While decades of study have made considerable progress in understanding how *Salmonella* causes disease, many challenges remain. Much is now understood about how *Salmonella* triggers its internalization within host cells and exacerbates inflammation. After the establishment of infection, *Salmonella* goes to considerable lengths to rein in the inflammation it initially enhanced to establish a long-term relationship with its host and, in some cases, to facilitate the systemic spread of infection [116]. In fact, attenuating inflammation appears to be key to allowing extraintestinal dissemination [11,116]. SopE promotes the initial ruffling response, whereas the slower degradation of SptP allows Rac and Cdc42 signaling to be subsequently shut down, which returns the cells towards homeostasis. In fact, *Salmonella* modulates the inflammatory response throughout the infection by manipulating a variety of distinct host pathways including NF-κB, NOD1 signaling, MAPK pathways, and inflammasome activation. More work is required to understand how spatiotemporal regulatory inputs are integrated by *Salmonella* to facilitate a balance between infectiousness and persistence. *Salmonella* has two strategies for intracellular survival and growth. Some *Salmonella* escapes the vacuole that enclose it and replicates extensively in the cytosol of infected epithelial cells [52,53,54,55]. For reasons that are not clear, some *Salmonella* remains within the vacuole and extensively modifies it to render it more conducive to bacterial growth. More is being understood on a regular basis about how *Salmonella* that remains within the *Salmonella*-containing vacuole can survive and proliferate within it by manipulating the endocytic pathway and intracellular trafficking events. Less is understood about how the pathogen grows within epithelial cells by escaping the vacuole that encloses it and proliferating within the cytosol. Both modes of intracellular survival and growth likely contribute significantly to *Salmonella* pathogenesis.

A layer of complexity that we are just beginning to appreciate the importance of is that there are many different naturally occurring alleles of each virulence gene that affect microbial pathogenesis in different, in some cases opposed ways. Virulence genes have typically been noted as simply being present or absent from a strain; however, in the case of SrfH and SpvD, different naturally occurring alleles can change the course of infection [10,148]. Expanding on these initial findings, a bioinformatics study that looked at type III effectors revealed that dominant alleles of ten genes were associated with systemic infections while dominant alleles of another ten genes were only present in strains that cause gastrointestinal disease [51]. Most virulence genes have dozens of naturally occurring alleles [51]. A great deal of work will be required to understand the effects of these alleles on various aspects of virulence. In the case of SrfH, interestingly, different naturally occurring alleles seem to have opposite effects on virulence, with one allele suppressing the systemic spread of infection and the other promoting it [9,10,117].

In addition to the possibility of different alleles of virulence genes having different effects, many proteins involved in virulence are believed to have overlapping functions. One report found that only five *Salmonella* pathogenicity island-2 effectors were necessary for bacterial survival and proliferation within macrophages, suggesting extreme functional redundancy [145]. However, in a different study using mice, 48 *Salmonella* pathogenicity island-1 and *Salmonella* pathogenicity island-2 effectors were individually replaced with DNA barcodes and the competitive index of each mutant was determined and compared with wild-type bacteria [191]. Surprisingly, mutating all but seven of the effectors affected microbial persistence, which argues against redundant functions [191]. Going forward, it will be important to determine the circumstances in which individual genes play a role in virulence.

Most virulence genes seem to have multiple, in some cases unrelated or even opposed functions within host cells. SopB, SopD, and SrfH are interesting examples of multi-function effectors, which display seemingly opposed activities. *Salmonella* regulates inflammatory signaling rather than simply stimulating it and inhibiting it. Interestingly, while SopB is well established to have proinflammatory effects by activating Cdc42, it also has anti-inflammatory effects with its phosphatidylinositol phosphatase activity, which ultimately inhibits the NLRC4 inflammasome [154]. Similarly, SopD is a dual function effector that stimulates both pro- and anti-inflammatory signaling by manipulating the Rab8 GTPase [42,151,152,153]. SopD inhibits Rab8 with its GTPase activating protein activity to stimulate inflammation but also activates anti-inflammatory signaling by displacing Rab8 from its cognate guanosine disassociation inhibitor. Thus, this effector has both agonistic and antagonistic activities upon the same host target to either augment or repress inflammation. One minor allele of the type III effector SrfH manipulates the host protein TRIP6 to facilitate the systemic spread of infected phagocytes [9,10]. This requires Gly103 instead of Asp103, which is more common [10]. SrfH Gly103 promotes the extraintestinal dissemination of infected phagocytes but also can suppress the deadhesion/migration of infected phagocytes with Cys178 [117]. How the opposed activities of these intriguing effectors are regulated to promote virulence is in need of further clarification.

A growing problem with *Salmonella* is multi-drug resistance. In fact, more than 60% of strains within the typhoidal serovars display such resistance [192]. Typhoidal *Salmonella* has acquired specific resistance mechanisms to ampicillin, chloramphenicol, and trimethoprim, leading to numerous outbreaks [192]. As a result, chloramphenicol was replaced with fluoro-quinolones and third generation cephalosporins as the first line drugs for typhoid fever [193]. There have been outbreaks of typhoid fever caused by strains resistant to ciprofloxacin and nalidixic acid that are now endemic in India and have been reported in the USA and UK, reflecting the emergence of a global public health problem [194]. Nearly intractable typhoid fever may occur soon because of the presence of a plasmid-borne integron in ciprofloxacin-resistant *S.* Typhi [194]. In fact, existing drugs target most *Salmonella* molecules considered ‘drugable’ [195,196]. Nearly all *S*. Typhimurium enzymes are not essential because of metabolic redundancies, and nearly all druggable microbial targets are interdicted with existing drugs [196]. Point mutations, such as those in *gyrA* and *parC* that confer resistance to ciprofloxacin, are very common. Thus, a challenge for the field is the development of anti-microbial drugs which the bacteria will be unable to quickly evolve ways to overcome. One possibility is the development of drugs that target the host factors that promote intracellular *Salmonella* proliferation. A global RNAi screen identified 252 such human-host-susceptibility factors for *S*. Typhimurium [197]. Silencing these genes did not adversely affect host cell health but impeded intracellular *S*. Typhimurium growth. Point mutations are not likely to confer resistance to drugs that target such host-susceptibility factors as this would presumably require large genetic changes.

## Figures and Tables

**Table 1:** Effectors that facilitate bacterial entry into host cells.

Virulence Factor	Secreted by SPI-1 or SPI-2	Cellular Target(s)	Function
SopE	+/−	Cdc42, Rac1, RhoG Caveolin-1	Rho GTPase exchange factor. Triggers membrane ruffling.
SopE2	+/−	Cdc42, Rac1	Similar to SopE.
SopB	+/−	Cdc42, RhoD, RhoB, RhoH, RhoJ, SNX9, SNX18	Interdicts inositol phosphatase signaling pathways.
SipA	+/−	Caspase-3, F-actin, T-plastin, syntaxin8	Regulates concentration, stability and polymerization of actin.

**Table 2:** Virulence factor distribution amongst *Salmonella* serovars.

	Ent	Ent iNTS	Tm	Tm iNTS	Dub	Chol	Gal	Pull	Typhi
SopE	+	+	+/−	−	+	−	+	+/−	+
SopE2	+	+	+	+	+	+	+	+	−
SopB	+	+	+	+	+	+	+	+	+
SipA	+	+	+	+	+	+	+	+	+
SopA	+	+	+	+	+	+	−	−	−
SopD	+	+	+	+	+	+	+/−	+/−	+
SrfA	+	+	+	+	+	+	+	+	+
SspH2	+	+/−	+	+/−	+/−	−	−	−	+
YdgT	+	+	+	+	+	+	+	+	+
RecA	+	+	+	+	+	+	+	+	+
CorA	+	+	+	+	+	+	+	+	+
AsmA	+	+	+	+	+	+	+	+	+
SteE	−	−	+	+	+	+	+/−	+	−
GogB	−	+/−	+	+	+	−	−	−	−
AvrA	+	+	+	+	+	−	+	+	−
GogA	−	−	+	+	−	−	−	−	−
PipA	+	+	+	+	+	+	+	+	+
GtgA	+	+	+	+	+	+	+	+	−
SseK1	+	+	+	+	+	+	−	+	−
SseK2	−	−	+	+	−	−	−	−	−
SseK3	+/−	+/−	+/−	+/−	+	+	−	−	−
SteA	+	+	+	+	+	+	+	+	+
SopD	+	+	+	+	+	+	+	+	+
SspH1	−	−	+/−	+/−	−	−	−	−	−
SptP	+	+	+	+	+	+	+	+	+
SpvB	+	+	+	+	+	+	+	+	−
SpvC	+	+	+	+	+	+	+	+	−
SpvD	+	+	+	+	+	+	+	+	−
SopF	+	+	+	+	+	+	+	+	+
SifA	+	+	+	+	+	+	+	+	+
SseF	+	+	+	+	+	+	+	+	+
SseG	+	+	+	+	+	+	+	+	+
SseJ	+	+	+	+	+	+	+	+	−
SseL	+	+	+	+	+	+	+	+	+
SrfH	+	+/−	+	+/−	+	+/−	−	−	−
SteC	+	+	+	+	+	+	+	+	+
PipB	+	+	+	+	+	+	+	+	+
PipB2	+	+	+	+	+	+	+	+	+
SopD2	+	+	+	+	+	+	+	+	−
GtgE	−	−	+	+	+	+	−	−	−

**Table 3:** Effectors that exacerbate inflammation.

Virulence Factor	Secreted by SPI-1 or SPI-2	Cellular Target(s)	Function
SopA	+/−	TRIM56, TRIM65	Enhances interferon-ß expression.
SopD	+/−	Rab8	Rab8 GAP and GEF.
SrfA	+/−	IRAK-1-toll interacting protein	Disassociates IRAK-1-toll interacting protein from IL-1R-associated kinase-1 to activate NFκB signaling.
SspH2 (dominant Typhimurium allele)	+/+	UbcH5-ubiquitin, SGT1, NOD1	Activates NOD1 signaling.

**Table 4:** Genes that regulate *Salmonella* growth within infected epithelial cell cytosol.

Bacterial Protein	Function	Effect
YdgT	Negative regulator of horizontally acquired genes.	Negatively regulates the expression of SPI-1, SPI-2 and SPI-5 genes. Promotes *Salmonella* growth within epithelial cell cytosol.
RecA	Promotes recombination.	Recombinatorial DNA repair. Promotes *Salmonella* growth within epithelial cell cytosol.
CorA	Mg2^+^ influx channel.	Promotes *Salmonella* growth within epithelial cell cytosol.
AsmA	Enhances IL-18 release from epithelial cells.	Enhanced inflammasome activation. Suppresses the growth of *Salmonella* within epithelial cell cytosol

**Table 5:** Virulence genes that are anti-inflammatory.

Virulence Factor	Secreted by SPI-1 or SPI-2	Cellular Target(s)	Function
SopE	+/−	PLCϒ and villin in addition to Cdc42, Rac1 and RhoG	Causes depolymerization of actin.
SopA	+/−	Inhibits Cdc42 activation	Limits the invasion process to avoid stimulating an immune response.
SteE/SarA/GogC	+/+	GSK3*α/β*, *STAT3*	Induces transcriptional change to an anti-inflammatory phenotype
GogB	+/+	SKP1, FBXO22	Inhibits NF-κB signaling.
AvrA	+/+	IκBα	Deubiquitinates IκBα to prevent its degradation which inhibits NF-κB activation
GogA	+/+	P65, RelB	Inhibits NF-κB signaling.
PipA	−/+	NF-κB p65	Inhibits NF-κB signaling.
GtgA	+/+	Class II NF-κBs	Inhibits NF-κB signaling.
SseK1	+/−	FADD, TRADD	Prevents TNFα-stimulated NF-κB signaling.
SseK2	ND/+	ND	Prevents TNFα-stimulated NF-κB signaling.
SseK3	ND/+	TRIM32, TRADD	Prevents TNFα-stimulated NF-κB signaling.
SteA	+/+	PI (4) P	SIF formation
SopD	+/+	Rab8	Not only a GAP but also a GEF for Rab8
SopB	+/−	Cdc42, Akt	In addition to activating Cdc42, it activates Akt, which leads to the inhibition of the NLRC4 inflammasome.
SspH1	+/+	PKN1	E3 ubiquitin ligase. Prevents NF-κB-dependent gene expression
SptP	+/−	Rac, Cdc42	GAP for Rac and Cdc42. Shuts the proteins off to limit ruffling after activation by SopB and SopE/SopE2
SpvB	ND/+	G-actin	Destabilizes the cytoskeleton. Inhibits NF-κB activity. Regulates NRF2 to alter iron homeostasis. Subverts host membrane trafficking through clathrin and AP-1
SpvC	+/+	P38, JNK, Erk1/2	Interdicts MAPK signaling.
SpvD	+/+	Xpo2	Prevents the activation of NF-κB regulated promoters

## Data Availability

No new data were created or analyzed in this study. Data sharing is not applicable to this article.
